# Distortions of lip size bias perceived facial attractiveness

**DOI:** 10.1098/rspb.2025.0202

**Published:** 2025-04-09

**Authors:** David Alais, Jacqueline Stephens, Jessica Taubert

**Affiliations:** ^1^ The University of Sydney, Sydney, New South Wales, Australia; ^2^ The University of Queensland, Saint Lucia, Queensland, Australia

**Keywords:** distortions, lips, bias, perceived, facial, attractiveness

## Abstract

Perceiving faces as attractive or not guides decisions to approach or date a person and can sway opinions in recruiting and legal proceedings. However, the mechanisms underlying facial attractiveness are not fully understood. While popular models of face recognition emphasize holistic processing, individuals often attempt to enhance their own attractiveness in feature-centric ways (cosmetic surgery, make-up, injectables). Here, we use a local feature manipulation (lip expansion/contraction) and show that it alters the perceived attractiveness of male and female faces. Females showed peak preference for expanded lips when viewing female faces; males showed peak preference for contracted lips when viewing male faces. Distortions of lip size therefore mostly influence own-gender attractiveness ratings. Next, we tested whether visual adaptation to expanded or contracted lips would bias subsequent attractiveness judgements, and found peak attractiveness shifted towards the adapted lip size (e.g. expanded lips were preferred following exposure to expanded lips). Viewing faces with artificially altered lip size therefore powerfully influences attractiveness judgements. Outside the laboratory, cosmetic procedures to increase lip size are popular. Our findings indicate that (i) lip plumping will mostly appeal to women rather than men (who prefer thinner lips), and (ii) exposure to expanded lips renormalizes attractiveness to a larger baseline and may lead to lip dysmorphia.

## 1. Introduction

The ability to perceive and discriminate among faces is essential for human survival and for a wide variety of social functions. Learning to perceive facial expressions and distinguish between familiar and unfamiliar faces is important for safety [1] and is central to social development. A quick glance at a face can reveal the sex, age, emotional state, engagement and attentional direction of that face [2]. One key aspect of face perception is attractiveness. Perceived attractiveness sways mate selection and platonic relationship choices [3,4], as well as social exchanges [3], and influences recruitment [5,6] and legal proceedings [7,8]. Perception of facial attractiveness is a highly flexible and adaptive function that is based on a spectrum of natural face variations [9,10] and has historically been free from processes that can drastically alter facial appearance. The recent advancement of modern cosmetic procedures, however, has meant that faces can now be readily changed. Such procedures are increasingly accessible and affordable, and are resulting in dramatically increased use of plastic surgery and injectables [11,12]. In particular, procedures that involve plumping the lips are rapidly rising in popularity in Western society.

Interest in beauty and body image is becoming more common and central in contemporary Western society [13]. There is increasing scrutiny of even the most minor details of facial appearance, and facial procedures have become more advanced and specialized to cater to various expectations associated with desirable facial characteristics [14,15]. One of the most popular techniques is lip plumping, and in the current study, we examine whether manipulating lip size in face images influences the attractiveness ratings they are given. To our knowledge, no studies have solely manipulated lip size to investigate the role of local features in face attractiveness. Indeed, most models of face perception emphasize holistic representations [16,17], and since local feature manipulations have been shown not to impact identity judgements [18], it is unclear whether local manipulations would be sufficient to impact facial attractiveness judgements. Here, we used a local distortion technique to expand and contract lips in linear steps [19]. Then we used these continua to test the impact of lip-specific distortions on facial attractiveness. After measuring the ratings given to male and female face images by male and female participants, we used an adaptation paradigm [19,20] to expose participants for a period to either expanded or contracted lips and test the impact of this exposure on subsequent attractiveness judgements. To preview the results, males gave peak ratings to male images with thin lips and females gave peak ratings to female images with expanded lips.

## 2. Results

**Figure 1.**
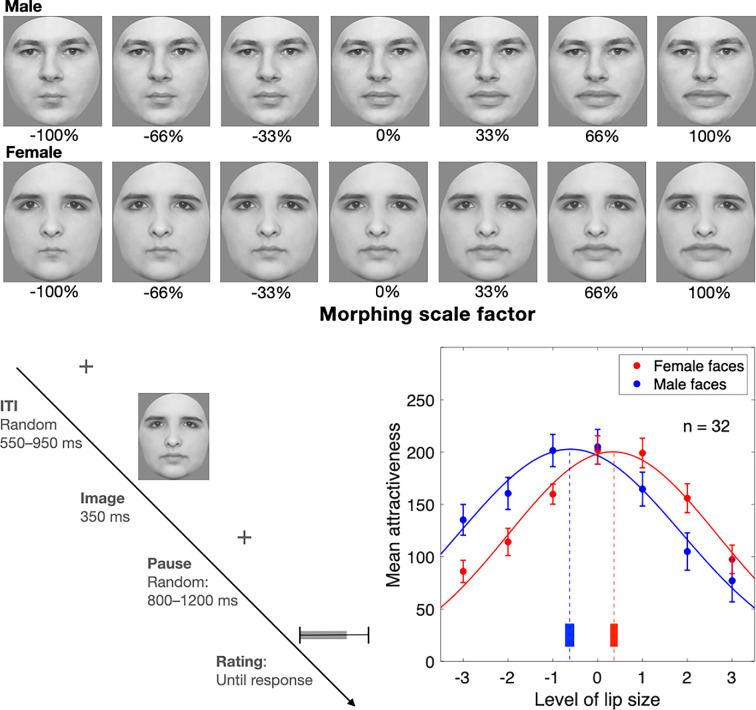
Top: example stimuli showing two identities and the seven levels of lip size. We used 24 identities (12 males, 12 females). Bottom left: the timing of a single trial. Participants adjusted a slider bar to rate attractiveness. Bottom right: attractiveness ratings plotted over lip size (*n* = 32; 16 males, 16 females). The curves are best-fitting Gaussians to group mean ratings of male (blue) and female (red) faces with ±1 standard error. Ratings curves for male and female faces are similar except for a pronounced and statistically significant lateral shift (*p* < 0.0001, Cohen’s *d* = 1.511). Vertical dashed lines indicate the Gaussian means and the vertical solid bars show the 95% confidence intervals based on 10 000 bootstraps. This establishes that the peak attractiveness of female faces occurs for slightly plumper lips and for male faces, it occurs for slightly thinner lips.

We collected data from 32 participants (16 males, 16 females) in experiment 1, who made attractiveness ratings of 24 face identities (12 males, 12 females) whose lips had been manipulated in size over seven levels, as illustrated in figure 1. Participants rated the faces six times each, and those ratings were averaged into a mean for a given participant’s rating of that identity/lip-size combination. The mean ratings data for the group are plotted in figure 1, with ratings of male and female faces plotted separately. The data showed a clear central tendency over the lip-size range and were fitted with Gaussian distributions so that the mean, standard deviation and amplitude parameters could be obtained and compared for each set of ratings. The Gaussian functions capture the pattern of data well (male faces: *r*^2^ = 0.983; female faces: *r*^2^ = 0.982) and reveal two very similar shaped distributions with a lateral offset. We tested for parameter differences by bootstrapping each participant’s data 10 000 times, refitting a Gaussian to the group mean ratings on every iteration and conducting bootstrap sign tests on the best-fit parameters. This revealed that the distribution means differed significantly (male faces, M = −0.611; 95% CI = [−0.703, −0.526]; female faces, M = 0.334; 95% CI = [0.256, 0.415], *p* < 0.0001), as did the standard deviations (male faces, M = 2.476; 95% CI = [2.371, 2.593]; female faces, M = 2.329; 95% CI = [2.229, 2.437], *p* = 0.0281). Amplitudes, however, were not significantly different (male faces, M = 202.814; 95% CI = [196.554, 209.107]; female faces, M = 200.311; 95% CI = [193.488, 206.988], *p* = 0.2964). The differences observed between the means establish that there is a strong bias (Cohen’s *d* = 1.511) in attractiveness ratings of female faces towards plumper lips and an opposing bias in attractiveness ratings of male faces towards thinner lips (see figure 1).

**Figure 2.**
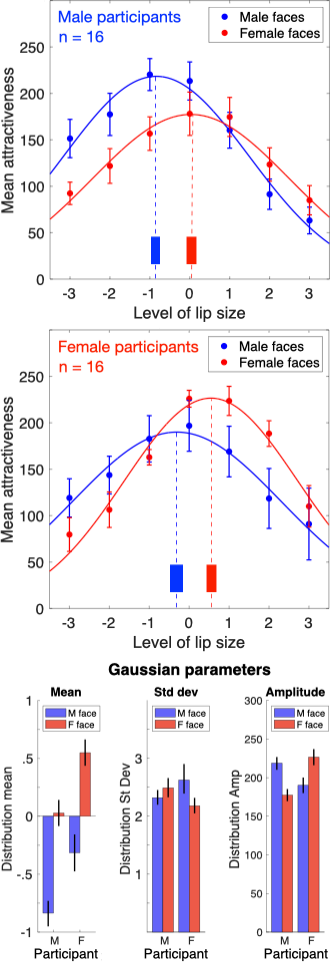
Top and middle panels: ratings for male and female faces, divided by male observers (top) and female observers (middle), with best-fitting Gaussian functions. Data points show group mean ratings with ±1 s.e.m. There are two clear results: (i) attractiveness ratings are higher within sex (e.g. males rating male faces) and (ii) the preference seen for females with plumper lips in the whole-group data (figure 1) is driven by female participants. The peak preference for females with plumper lips is present only in the female participants (middle panel). Male participants show a peak preference that is not significantly different from 0 (i.e. unmorphed lips). Vertical dashed lines indicate the Gaussian means and the vertical solid bars show the 95% confidence intervals. Bottom panel: parameters for the best-fitting Gaussian functions fitted to the data in the top and middle panels, with error bars showing 95% confidence intervals. The distribution means (left) show that peak attractiveness for males rating female faces is not significantly different from zero (i.e. neutral, unmorphed lips), whereas ratings of females by females show a peak shift towards plumper lips. Data for male faces show the inverse pattern: male participants show a peak attractiveness for thinner lips than is seen in female participants.

Given the overall preference for female faces with plump lips and for male faces with thin lips, we next divided the group into male (*n* = 16) and female (*n* = 16) participants to test whether ratings differed for own-gender stimuli. Figure 2 shows the results of the same analysis in figure 1, but split by the sex of the observer. The upper panel shows data from male participants and the lower panel shows data from female participants. The preference for thinner lips in male faces is again present (upper panel, leftward shift of the blue curve) but splitting the participant group by sex has revealed that the effect is even stronger in male observers compared with female observers. This is evident in the distribution’s higher amplitude, indicating higher overall attractiveness ratings for males rating male faces. We tested the differences between these distributions using the same bootstrapping procedure described above to compute population means and 95% confidence intervals (figure 2, bottom panel). This analysis confirmed that for male observers, the distribution means differed significantly (male faces, M = −0.838; 95% CI = [−0.941, −0.741]; female faces, M = 0.027; 95% CI = [−0.083, 0.137], *p* < 0.0001, Cohen’s *d* = 1.585), as did the amplitudes (male faces, M = 218.543; 95% CI = [210.683, 226.485]; female faces, M = 177.396; 95% CI = [170.232, 184.734], *p* < 0.0001). There was also a difference between standard deviations that trended very close to significance (male faces, M = 2.324; 95% CI = [2.212, 2.448]; female faces, M = 2.484; 95% CI = [2.339, 2.646], *p* = 0.0508).

The preference seen in figure 1 for female faces with plump lips (rightward shift of the red curve) is clearer in the middle panel of figure 2. For female observers, overall attractiveness ratings are higher for female faces than for male faces. Tests of the parameter differences for female observers confirmed a significant difference for all three distribution parameters: mean (male faces, M = −0.311; 95% CI = [−0.468, −0.166]; female faces, M = 0.548; 95% CI = [0.433, 0.664], *p* < 0.0001, Cohen’s *d* = 2.189), standard deviation (male faces, M = 2.621; 95% CI = [2.431, 2.843]; female faces, M = 2.174; 95% CI = [2.043, 2.320], *p* < 0.0001) and amplitude (male faces, M = 190.045; 95% CI = [180.209, 200.012]; female faces, M = 226.738; 95% CI = [215.028, 238.753], *p* < 0.0001).

The results are summarized in the bottom panel, which plots the parameters from the best-fitting Gaussian ratings functions, with error bars showing 95% confidence intervals. All three parameters show interesting effects when the sex of the observer and stimuli match. The Gaussian means show that the preference for males with thinner lips and females with plumper lips is even stronger for own-gender attractiveness judgements. The congruence between the observer and image also produced rating distributions with reduced standard deviations and higher amplitudes. Observers thus give their highest and most precise ratings to face stimuli representing their own sex. To quantify this, the grand average attractiveness ratings for male observers were 153.9 when rating male faces and 133.2 for female faces. For female observers, the grand averages were 156.7 for female faces and 145.8 for male faces.

Next, we ran adaptation experiments in which faces with distorted lips were presented for longer exposure periods sufficient to produce face adaptation and after-effects [21,22]. We compared two different face adaptors: faces with lip size either expanded or contracted by two steps (see stimuli in figure 1 with ± 66% change in lip size). There are many reports of face after-effects, and it has been a useful paradigm for evaluating how previous experience and visual input influences the recognition of different facial attributes [20]. For example, adaptation to faces has been previously shown to influence subsequent facial judgements [17], but these reports have all employed manipulations of entire faces, such as inversion [23] or negation [24]. Would adaptation to faces with artificially enlarged or contracted lips modulate attractiveness ratings? If so, would such experience reshape the perceived attractiveness of all faces or merely the adapted identity? We asked these questions by comparing the size of the lip-distorted face after-effect on the same or different identities in the test phase.

**Figure 3.**
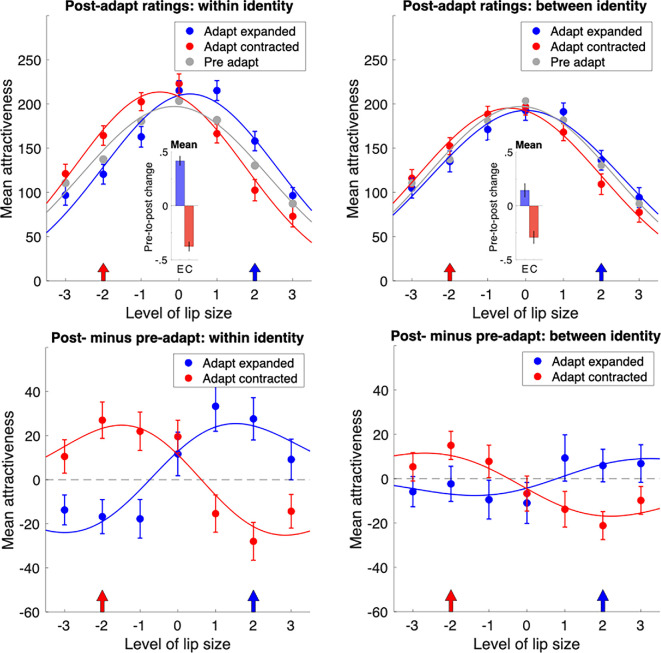
Attractiveness ratings after adaptation to altered lip size. Arrows on the *x*-axis show the adapted lip size. The left panels show data for adaptation and testing within the same identity; the right panels show between-identity data. After adaptation, attractiveness distributions shift towards the value of the adapted lip level. Insets show the change in the distribution mean from the pre-adaptation baseline (grey curves) with 95% confidence intervals. Bottom panels: to better reveal the pre- to post-adaptation change, we plot a difference-of-Gaussians (DoG) model using the parameters from the panels above. The amplitudes of the DoG fits show that adaptation within identity modulates attractiveness ratings by ±24 points for expanded lips and ±25 points for contracted lips. Between-identity results are qualitatively similar but weaker, with modulations of ±8 and ±14 points for expansion and contraction, respectively.

Data from the lip adaptation experiment are shown in figure 3, with the left panels showing results for adaptation and testing within the same identity and the right panels showing between-identity results. The rating distributions for unadapted faces provide a baseline and are shown in grey. Consistent with previous face adaptation studies and the theory of adaptation shifting the norm of a space or dimension [10,20], both upper panels show clear shifts in the distribution of attractiveness ratings towards the value of the adaptor, indicated by the arrows on the *x*-axis. For the within-identity condition, there was a significant pre- to post-adaptation shift in the direction of expanded lips after expanded adaptation (M = 0.417; 95% CI = [0.371, 0.463], *p* < 0.0001) and a similar shift towards contracted lips after contracted adaptation (M = −0.376; 95% CI = [−0.422, −0.3313], *p* < 0.0001). The adaptation shifts in the between-identity condition followed the same pattern but were smaller overall: adaptation to expanded lips (M = 0.146; 95% CI = [0.082, 0.212], *p* < 0.0001); adaptation to contracted lips (M = −0.293; 95% CI = [−0.354, −0.232], *p* < 0.0001). The rating data after adaptation remain well described by a Gaussian, and the shift is captured by the change in distribution mean from pre- to post-adaptation, as shown by the insets in the upper panels with error bars showing 95% confidence intervals.

The lower panels of figure 3 show the difference between the pre- and post-adaptation data, with the continuous lines showing a difference-of-Gaussian (DoG) model, taking the best-fitting parameters from the pre- and post-adaptation fits in the upper panels. The amplitude of the DoG functions quantifies the size of the adaptation effects. For the within-identity data (left panels), adaptation to expanded lips modulated attractiveness ratings by ±24 points and adaptation to contracted lips modulated attractiveness by ±25 points. Based on the ratings in the pre-adaptation data spanning a range of 116 points, these modulations represent shifts in attractiveness ratings of 22% following adaptation. When different identities were used for adaptation and testing (bottom right), the results were qualitatively similar, but the adaptation-related modulations in attractiveness ratings were smaller: ±8 points for adaptation to expanded lips, ±14 points for adaptation to contracted lips (average modulations of about 9%). Overall, the adaptation-related shifts in peak attractiveness rating occur equivalently for adaptation to expansion and contraction, although the effect is stronger when adaptation and testing occur within the same face.

**Figure 4.**
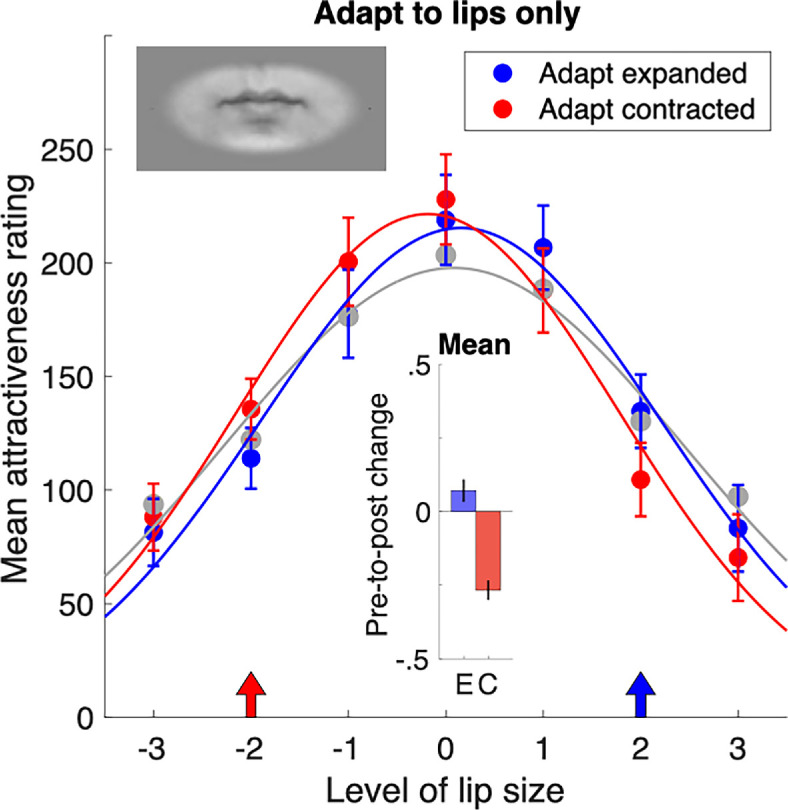
Attractiveness ratings after adaptation to expanded and contracted lips presented in isolation. Arrows on the *x*-axis show the adaptor lip size. The left panels show data for adaptation and testing within the same identity; the right panels show between-identity data. After adaptation, attractiveness distributions shift towards the value of the adapted lip level. Insets show the change in the distribution mean from the pre-adaptation baseline (grey curves) with 95% confidence intervals. Bottom panels: to better reveal the pre- to post-adaptation change, we plot a DoG model using the parameters from the panels above. The amplitudes of the DoG fits show that adaptation within identity modulates attractiveness ratings by ±24 points for expanded lips and ±25 points for contracted lips. Between-identity results are qualitatively similar but weaker; with modulations of ±8 and ±14 points for expansion and contraction, respectively.

Finally, we looked at whether adaptation to lips alone would be sufficient to shift attractiveness ratings of test faces. The theory of holistic processing for face perception posits that isolated lips would be represented separately from whole faces with lips [25,26]. If this is the case, and holistic processing underwrites facial attractiveness, then adapting to isolated lips should have no impact on the subsequent judgements of whole faces. To make the lips-only stimuli, we masked the faces with a soft-edged oval mask, which isolated the lips and had a size sufficient to encompass the largest lips in our stimulus set. No other facial features were visible (see inset, figure 4). The results of lips-only adaptation are plotted in figure 4 and show that there is a significant post-adaptation shift in attractiveness ratings distributions. As in figure 3, the effect occurred in both directions, with exposure to expanded lips (blue arrow) shifting peak attractiveness towards plumper lips (M = 0.072; 95% CI = [0.036, 0.108], *p* < 0.0001) and exposure to contracted lips (red arrow) shifting peak attractiveness towards thinner lips (M = −0.267; 95% CI = [−0.301, −0.233], *p* < 0.0001). Notably, the shifts are smaller than those that occurred for adaptation to distorted lips in a whole-face context tested on within-identity test patterns, although they are similar to the more modest effects obtained for between-face adaptation (compare upper panels, figure 3). These results imply that lip size is encoded separately from the whole face or other local features.

## 3. Discussion

Here, we used psychophysics to investigate the impact of lip-specific distortions on facial attractiveness. In figure 1 the data reveal that female faces are rated as more attractive when the size of their lips has been expanded. In contrast, male faces are rated as more attractive when the size of their lips has been contracted. These preferences are consistent with lip size and width being sexually dimorphic traits that can diagnose genetic sex [27] as well as reproductive health [28] and fecundity [3,29,30]. However, a deeper level of analysis reveals that the lip size preferences we report were unexpectedly driven by own-gender biases.

Our data show that, on average, female participants gave higher attractiveness scores to female faces, whereas male participants gave higher attractiveness scores to male faces. The results of previous studies have suggested that when we are asked to judge facial attractiveness, our scores are anchored by our own attributes [31,32]. Therefore, it seems possible that we are more confident and more accurate when tasked with evaluating the attractiveness of faces that belong to our own social groups, such as our own gender and own race [33]. Importantly, in figure 2, we also show that male participants had a strong preference for male faces with thin lips. Conversely, female participants had a strong preference for female faces with plump lips. In other words, our data suggest that costly cosmetic procedures that increase female lip size, such as injectables and lip-enhancing surgeries, may not appeal to men.

In this study, we also investigated the impact of exposure to distorted lips on facial attractiveness. Figure 3 provides clear evidence that when participants were exposed to a new lip size, that lip size became the new norm. Visual adaptation is known to impact a wide variety of facial attributes, including identity [20,34], expression [35–37] and gender [35,36,38,39]. This process of renormalization is commonly believed to be highly adaptive and beneficial, allowing the visual system to stay calibrated with the current environment and sensitive to important changes in visual input. However, in the context of facial attractiveness, it is possible that this foundational visual process, which works outside of our awareness, is maladaptive. Exposure to distorted lips that have been artificially expanded beyond biological limits could initiate an iterative process whereby only further expansion to even plumper lips will be perceived as attractive to an adapted observer. Ultimately, such a process would culminate in lip dysmorphia.

Similar mechanisms are thought to underlie body dysmorphia. Previous studies have also used visual adaptation to show that the most attractive and most normal-appearing bodies become thinner after prolonged exposure to thin bodies [40–42]. The current data show that the maladaptive influence of visual adaptation is not limited to bodies. Here, we provide empirical evidence that the perception of discrete facial features is also subject to visual adaptation. It follows that, in the real world, when people are seeking to permanently modify their appearance with surgery, more safeguards are required because, over time, they will visually adapt to the new shape or size of their modified features, and this will likely normalize their definition of attractiveness and motivate further surgery.

To establish a connection between visual adaptation and the development of facial dysmorphia in real-world situations, it is important to investigate whether the effects of adaptation can transfer between different faces. Here, we found that the re-normalizing effect of lip-size adaptation does occur across different facial identities, although it is considerably stronger within the same identity (figure 3). This cross-identity observation suggests that exposure to distorted lip sizes will influence how we perceive lip size in all faces, including our own. Further, these findings also indicate that the neural mechanisms responsible for encoding lip size in faces overlap to some extent across different facial identities, but not completely [43,44]. Additionally, the results of the adaptation experiment provide new insights into the origin of the own-gender biases observed in facial attractiveness judgements (figure 2) by suggesting that different preferences for male and female lip size could reflect differences in male and female lip size in the real world that are amplified by greater exposure and attention to own-gender faces (including one’s own face). This idea warrants further exploration in future research.

In a final experiment, we found that adaptation to lips alone produced significant shifts in attractiveness ratings. This indicates that lips do not need to be in the context of a whole face to modulate subsequent attractiveness judgements. Further, this implies that lips are represented on their own stimulus dimension, separate from the whole face context. This is consistent with very recent evidence showing that facial features, such as lips and eyes, are represented separately in the macaque visual cortex [45–47]. There have also been several reports showing that increases in familiarity increase feature-based processing strategies during face recognition tasks [48,49]. Together with the current findings, these recent findings challenge the long-held idea that faces are processed holistically. Instead, it appears that, under certain circumstances, our brains can rely on information from individual facial features. For instance, when evaluating attractiveness, we may evaluate feature-specific details rather than the entire face. This could explain why people use strategies like injectables, make-up or tattoos to address perceived deficiencies in their appearance.

Overall, this study uncovered clear evidence that local information about a single feature, specifically the lips, can strongly influence facial attractiveness. Interestingly, lip size interacts with the stimulus gender such that male faces are rated as more attractive when lips are thinner, whereas female faces are rated as more attractive when lips are plumper. Additionally, we have shown that these biases are adaptable, with norms resetting after exposure to distorted lip sizes. Although the factors that contribute to facial attractiveness and estimates of beauty remain largely unknown [3], these findings indicate that discrete local features can contribute substantially. These findings therefore open new avenues for understanding the representation of faces and the mechanisms underlying attractiveness.

## 4. General methods

### (a) Participants

Participants were recruited with no restrictions from psychology students at the University of Sydney, who participated for course credit. There were 32 participants in total (16 female, 16 male).

### (b) Stimuli

Experiment 1: 24 face identities (12 female, 12 male) were used as stimuli in the rating experiment, drawn from a set used in previous studies [50–52]. All images depicted neutral expressions and were forward-facing, with external cues to facial identity (e.g. hair, ears and neck) removed using an oval-shaped mask. The oval window had a fixed height of 6.4° of visual angle and a width that was adjusted based on the shape of each face. The luminance and root-mean-square contrast of all stimuli were standardized to match the mean of the image set. For each identity, seven levels of lip distortion were created: an original with natural dimensions, plus three with magnified lip distortions and three with minified lip distortions (see examples in figure 1). This produced 168 face stimuli (24 identities × 7 lip levels).

Experiment 2: the same stimuli were used in the adaptation study. For the adaptation phase, each participant saw three of the 24 identities presented with lips that were either moderately expanded (+2 levels) or moderately contracted (−2 levels). Adaptation lasted for 15 s initially, followed by 4 s periods of top-up adaptation for subsequent trials. Test stimuli were presented for 350 ms, with a 1 s blank screen between adaptation and testing. For the test phase, all seven lip sizes for a given identity were used, each tested four times.

### (c) Design

Experiment 1: the 168 face stimuli were presented in separate blocks of male and female faces (84 faces each: 12 identities × 7 lip sizes), with the male/female order counterbalanced across participants. Faces were briefly presented (350 ms) and rated for attractiveness. Within blocks, the order of trials was completely randomized so that any repetition effect could not systematically bias the data. The full set of 168 stimuli was presented and rated five times (840 trials in total). Half of the male and female participants viewed the female images first, and the other half viewed the male images first.

Experiment 2 was an adaptation experiment. As a pre-adaptation baseline, participants completed a brief attractiveness rating experiment similar to experiment 1. Participants adapted to three identities (each presented with moderately expanded or contracted lips) and were then tested with all seven levels of lip size. Across all participants, every identity was used as an adaptor. We also compared within-identity and between-identity adaptation: for half of the subjects (8 males, 8 females), the seven test stimuli had the same identity as the adaptor, and for the other half, the test had a different identity. Baseline ratings were made for all faces seen by a given participant.

### (d) Procedure

Experiment 1: attractiveness ratings. Participants were instructed to fixate on a small cross at the screen centre during trials, which remained visible throughout the experiment. Faces were presented for 350 ms in locations that jittered around the fixation point (to avoid local adaptation effects and predictable feature locations). After a 1.25 s blank screen, an adjustable slider bar was presented to indicate attractiveness ratings (adjusted using the left/right arrows on the keyboard). After a further 750 ms blank interval, the next face appeared.

Experiment 2: adaptation to distorted lips. The pre-adaptation ratings were conducted as in experiment 1. The adaptation phase involved a 15 s initial exposure to an adapting face (lip level −2 or +2) followed by an attractiveness rating of one of the seven test faces. After the initial adaptation to a given adaptor face, top-up exposures were 4 s duration. All face locations were jittered in the location around the fixation on every presentation (adaptor and test stimuli). The adaptation and test faces were either the same (within-identity condition) or different (between-identity condition).

## Data Availability

Data and code are publicly available at OSF [53].
